# Stimulating β-Cell Regeneration by Combining a GPR119 Agonist with a DPP-IV Inhibitor

**DOI:** 10.1371/journal.pone.0053345

**Published:** 2013-01-29

**Authors:** Ansarullah, Yan Lu, Martha Holstein, Brittany DeRuyter, Alex Rabinovitch, Zhiguang Guo

**Affiliations:** 1 The Sanford Project, Children Health Research Center, Sanford Research/USD, Sioux Falls, South Dakota, United States of America; 2 Department of Pediatrics, Sanford School of Medicine, University of South Dakota, Sioux Falls, South Dakota, United States of America; 3 Department of Surgery, Sanford School of Medicine, University of South Dakota, Sioux Falls, South Dakota, United States of America; Broad Institute of Harvard and MIT, United States of America

## Abstract

**Background:**

Activating G-protein coupled receptor 119 (GPR119) by its agonists can stimulate glucagon like peptide-1 (GLP-1) release. GLP-1 is rapidly degraded and inactivated by dipeptidylpeptidase-IV (DPP-IV). We studied the efficiency of combining PSN632408, a GPR119 agonist, with sitagliptin, a DPP-IV inhibitor, on β-cell regeneration in diabetic mice.

**Materials & Methods:**

Diabetes in C57BL/6 mice was induced by streptozotocin. PSN632408 and sitagliptin alone or in combination were administered to diabetic mice for 7 weeks along with BrdU daily. Nonfasting blood glucose levels were monitored. After treatment, oral glucose tolerance test (OGTT), plasma active GLP-1 levels, β-cell mass along with α- and β-cell replication, and β-cell neogenesis were evaluated.

**Results:**

Normoglycemia was not achieved in vehicle-treated mice. By contrast, 32% (6 of 19) of PSN632408-treated diabetic mice, 36% (5 of 14) sitagliptin-treated diabetic mice, and 59% (13 of 22) diabetic mice treated with PSN632408 and sitagliptin combination achieved normoglycemia after 7 weeks treatment. Combination therapy significantly increased plasma active GLP-1 levels, improved glucose clearance, stimulated both α- and β-cell replication, and augmented β-cell mass. Furthermore, treatment with combination therapy induced β-cell neogenesis from pancreatic duct-derived cells.

**Conclusion:**

Our results demonstrate that combining a GPR119 agonist with a DPP-IV inhibitor may offer a novel therapeutic strategy for stimulating β-cell regeneration and reversing diabetes.

## Introduction

Progressive impairment of pancreatic β-cell function and decline in β-cell mass result in relative or absolute insulin deficiency and hyperglycemia, the primary basis of all diabetic manifestations [1], [2], [3]. Therefore, strategies that can induce β-cell regeneration have the potential to cure diabetes.

Glucagon like peptide- 1 (GLP-1) is released from the intestinal enteroendocrine L cells in response to nutrient ingestion. GLP-1 exerts pleiotropic actions in pancreatic islets that include stimulating glucose-dependent insulin secretion from β-cells, suppressing glucagon release from α-cells, enhancing β-cell proliferation, and preventing β-cell apoptosis [4], [5]. However, GLP-1 is rapidly degraded and inactivated by dipeptidylpeptidase-IV (DPP-IV), a serine protease present in soluble form in circulation. Thus, inhibition of DPP-IV leads to an increase in circulating levels of endogenous bioactive GLP-1 [6], [7]. DPP-IV inhibitors, such as sitagliptin, play a major role in preventing degradation of endogenous active GLP-1 and are being assessed extensively in clinical settings for their long-term efficacy in improving β-cell function in humans with type-2 diabetes mellitus [8], [9]. At present, DPP-IV inhibitors are the only agents in clinical use that increase endogenous GLP-1 levels [10], [11].

Islet β cells express several G-protein coupled receptors (GPR), one of which is the GLP-1 receptor and another one is GPR119 [12], which is expressed predominantly in pancreatic β cells and intestinal enteroendocrine L cells. GPR119 expression has been demonstrated in isolated islets and mouse insulinoma cell lines, indicating specific expression in β-cell lineage [13]. GPR119 agonists enhance glucose-dependent insulin secretion and improve glucose tolerance in wild-type mice, but not in GPR119 knockout mice [14]. Activation of GPR119 by endogenous ligands, like oleoyl lysophosphatidylcholine and oleoylethanol amide, or small molecule agonists, leads to accumulation of intracellular cAMP and further GLP-1 and insulin release [12], [13], [14], [15], [16].

PSN632408, a selective small molecular GPR-119 agonist, can increase intracellular cAMP levels in a GPR-119 dependent manner and reduce food intake and body weight gain in rodents [15]. Recently, we demonstrated that PSN632408 can stimulate β-cell replication in mouse islets *in vitro* and *in vivo* and can improve islet graft function and plasma active GLP-1 levels were elevated by this GPR-119 agonist [17]. Therefore, PSN632408 may improve islet function and stimulate β-cell regeneration through either direct activation of β cells or indirectly by stimulating GLP-1secretion.

We hypothesized that combining a GPR119 agonist with a DPP-IV inhibitor could potentially improve the therapeutic effectiveness of GLP-1 by stimulating its release through activating GPR119 on intestinal enteroendocrine L cells while simultaneously preventing its degradation by inhibiting DPP-IV. To test this hypothesis, we used streptozotocin (STZ), a β-cell specific toxin, to induce diabetes in a mouse model of insulin-deficient diabetes and to assess the efficiency of the GPR119 agonist, PSN632408, and the DPP-IV inhibitor, sitagliptin, alone and in combination on improving pancreatic β-cell function, stimulating β-cell regeneration, and reversing diabetes.

## Materials and Methods

### Animals

Eight week-old male C57BL/6J mice were purchased from Jackson Laboratories (Bar Harbor, ME, USA) and were housed in a specific-pathogen free animal facility at Sanford Research/USD. All experiments were performed in accordance to the protocol approved by the Sanford Research/USD Institutional Animal Care and Use Committee (#25-01-14B).

### Diabetes Induction and Glucose Measurement

C57BL/6 mice were injected with STZ (Sigma-Aldrich, St Louis, MO, USA) intraperitoneally to induce diabetes. Blood glucose was measured by the tail vein sampling method using Bayer Contour™ Glucometer (Bayer HealthCare, Tarrytown, NY, USA). Diabetes was diagnosed when the non-fasting blood glucose was >400 mg/dL on two consecutive measurements. Only diabetic mice with blood glucose levels between 400–500 mg/dL on two consecutive measurements were used for treatment. The day of achieving normoglycemia in diabetic mice was defined as the time when the nonfasting blood glucose level was <200 mg/dL on two consecutive days of measurements without any insulin treatment.

### Treatment

Diabetic mice were treated by gavage with vehicle (PBS), PSN632408 (Cayman Chemicals, Ann Arbor, MI, USA) at 10 mg/kg or sitagliptin (Merck, Whitehouse Station, NJ, USA) at 60 mg/kg, either alone or in combination, daily for 7 weeks. In addition, all diabetic mice were treated with 1.0 U of insulin daily. Insulin treatment was stopped if the blood glucose level was <200 mg/dL. To label replicating cells, all mice were injected intraperitoneally with bromodeoxyuridine (BrdU) at 100 mg/kg daily for 7 weeks. After treatment, mice underwent oral glucose tolerance tests (OGTT) and then were euthanized to collect blood samples and pancreases by intraperitonally injection of an overdose of Avertin (700 mg/kg).

### OGTT

At the end of the treatment period, mice with normoglycemia underwent OGTT. Mice were fasted overnight and blood glucose levels were measured by tail vein sampling. A single dose of PSN632408 10 mg/kg, or sitagliptin 60 mg/kg, or PSN632408 and sitagliptin combination, or vehicle was administered by gavage. After 30 minutes, glucose at 2 g/kg of body weight was given by gavage. Blood glucose levels were determined at −30, 0, 30, 60, 90 and 120 min after glucose administration. The results are expressed as integrated area under the curve (AUC_0–120_) for glucose, calculated by the trapezoid rule [AUC = (C1+ C2)/2×(t_2_−t_1_)] and changes in glucose concentrations during OGTT are expressed as AUC_glucose_ (mg/dL per min).

### Immunofluorescence and Confocal Microscopy

Five micron thick serial pancreas sections were mounted on lysine coated slides (Fisher Scientific, Pittsburgh, PA, USA). For immunohistochemical detection of BrdU-incorporating nuclei, DNA was first denatured to expose the antigen by incubating the tissue sections in 1N HCl for 45 minutes at 37°C. The sections were rinsed three times for 5 minutes each in PBS and then were incubated with primary antibodies: rat anti-BrdU monoclonal antibody (1∶200) purchased from Accurate Chemicals (Westbury, NY, USA), and guinea pig anti-insulin (1∶200), rabbit anti-glucagon (1∶200), rabbit anti-amylase (1∶200), rabbit anti-cytokeratin-19 (CK-19, 1∶200), and rabbit anti-Ki67 (1∶200) purchased from Abcam (Cambridge, MA, USA). An antigen unmasking step was performed for 30 minutes in a pressure cooker using rodent decloaker solution (Biocare Medical, Concord, CA, USA). Non-specific blocking was performed using 4% normal donkey serum (Jackson ImmunoResearch Laboratories, West Grove, PA, USA). Next, the labeled sections were washed with PBS (3 times for 5 minutes each) and secondary antibody incubation was carried out in the dark for 45 minutes at 37°C. Fluorochrome-conjugated secondary antibodies Alexa-Fluor 488, Alexa-Fluor 546 or Alexa-Flour 647 F(ab’)_2_ (Jackson ImmunoResearch Laboratories) were used at 1∶200 dilution. DAPI was used to visualize nuclei. Negative controls were run where the primary antibodies were omitted. Tissue sections were washed in calcium and magnesium-containing PBS and mounted with anti-fade mounting medium Vectasheild (Vector Laboratories, Burlingame, CA, USA). Nikon TIRF, a laser scanning confocal microscope, was used with a 63X1.4NA pan Apochromat objective with optical Z sections taken at ∼0.8 microns. Magnification, laser and detector gains, and pinhole settings were set below saturation and were identical across samples.

### Pancreatic β-Cell Mass, Replication, and Neogenesis

To evaluate β-cell mass, pancreas sections from 4 to 6 mice of each group were analyzed for insulin area with NIS elements imaging software (Nikon Instruments Inc, Melville, NY, USA) using the lasso tool to select individual islets. β-cell mass (mg per pancreas) was calculated by multiplying relative insulin-positive area (the percentage of insulin positive area over total pancreas area) by pancreas weight. To evaluate β-cell replication, the ratio of insulin/BrdU/DAPI co-positive cells or insulin/Ki67/DAPI co-positive cells over the total insulin positive cells in islets in pancreas sections from 4 to 6 mice of each group was calculated. To evaluate β-cell neogenesis, the ratio of ducts with one or mare insulin/CK-19/DAPI co-positive cells over the total number of ducts per pancreas was calculated.

### Pancreatic α-Cell and Acinar Cell Replication

To determine α-cell replication, the ratio of all glucagon/BrdU/DAPI co-positive cells over the total number of glucagon positive cells was calculated. Acinar cell replication was calculated as the ratio of amylase/BrdU/DAPI co-positive cells over the total number of amylase positive cells.

### Plasma Active GLP-1 Assay

Blood samples from treated mice were collected at 30 minutes post-treatment with vehicle, PSN632408 10 mg/kg, or sitagliptin 60 mg/kg, or PSN632408 and sitagliptin combination (n = 7 to 8 per group). To preserve active GLP-1, a DPP-IV inhibitor (10 µl per mL of blood) was pre-added to the blood collection tubes and the capillary collection tubes were also coated with DPP-IV inhibitor. Active GLP-1 in plasma was measured by using GLP-1 a ELISA kit (Millipore Corporation, Billerica, MA) that is specific for the GLP-1 (7–36) amide form that represents the majority of circulating biologically active GLP-1.

### Statistics

Statistical evaluation of the data was done by one way Analysis of Variance followed by Bonferroni’s multiple comparison tests. The results are expressed as mean ± SEM using Graph Pad Prism version 5.0 (Graph Pad Software, San Diego, CA, USA). A value of *P*<0.05 was considered statistically significant.

## Results

### PSN632408 and Sitagliptin Reversed Diabetes after 7 Weeks of Treatment

**Figure 1:**
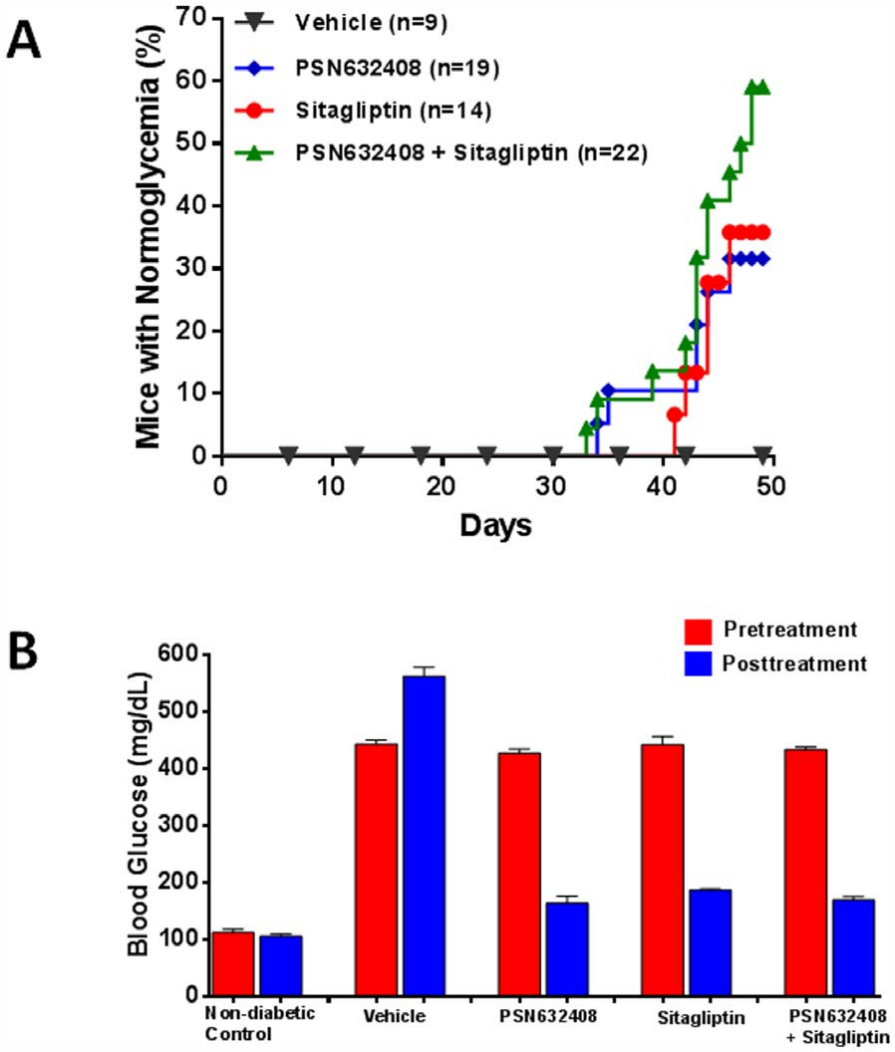
(A) Percentage of diabetic mice achieving normoglycemia after 7 weeks of treatment with vehicle, PSN632408, sitagliptin, and PSN632408 combined with sitagliptin. Normoglycemia was not achieved in vehicle-treated diabetic mice. By contrast, 32% (6 of 19) PSN632408-treated mice, 36% (5 of 14) sitagliptin-treated mice, and 59% (13 of 22) PSN632408 plus sitagliptin-treated mice achieved normoglycemia between 6 and 7 weeks (P<0.05, vs. PSN632408 alone or sitagliptin alone). **(B)** Pretreatment and posttreatment blood glucose levels in diabetic mice with restored normoglycemia (nonfasting blood glucose level <200 mg/dL) after treatment for 7 weeks. Posttreatment blood glucose levels were significantly reduced compared with pretreatment blood glucose levels in these mice (P<0.01).

Diabetic mice with blood glucose levels between 400 to 500 mg/dL were treated for 7 weeks. Normoglycemia was not achieved in vehicle-treated diabetic mice. None of the mice achieved normoglycemia in any treatment group during the first 4 weeks of treatment. However, 32% (6 of 19) PSN632408-treated diabetic mice and 36% (5 of 14) sitagliptin-treated diabetic mice achieved normoglycemia after 7 weeks of treatment (Figure 1A). In the mice treated with PSN632408 and sitagliptin combination, 59% (13 of 22) achieved normoglycemia after 7 weeks treatment (p<0.05, vs. PSN632408 alone or sitagliptin alone). In these mice with restored normoglycemia, the mean non-fasting blood glucose level before treatment was 427±7 mg/dL in PSN632408-treated mice, 428±9 mg/dL in sitagliptin-treated mice and 433±5 mg/dL in PSN632408 plus sitagliptin-treated mice. After treatment, the mean non-fasting blood glucose level was reduced to 163±12 mg/dL in PSN632408-treated mice, 186±2 mg/dL in sitagliptin-treated mice and 168±6 mg/dL in PSN632408 plus sitagliptin-treated mice (Figure 1B). Thus, PSN632408 and sitagliptin combination can improve the efficacy of either PSN632408 alone or sitagliptin alone on reversing diabetes.

### PSN632408 and Sitagliptin Treatment Increased Plasma Active GLP-1 Levels

**Figure 2:**
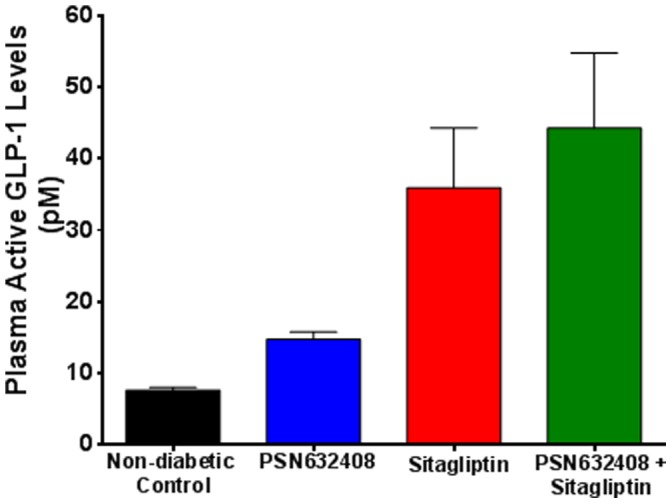
Active GLP-1 levels in plasma of mice 30 minutes after treatment with PSN632408, sitagliptin, or PSN632408 and sitagliptin combination (n = 7 to 8 mice per group). Plasma GLP-1 levels were significantly higher in mice treated with PSN632408 and sitagliptin combination than in mice treated with PSN632408 alone, (p<0.05).

At 30 minutes after treatment, plasma active GLP-1 levels were significantly higher in mice treated with PSN632408, sitagliptin, and PSN632408 plus sitagliptin compared with non-diabetic control mice (Figure 2). Plasma active GLP-1 levels were 7.6±0.4 pmol/L in untreated control mice, 14.8±1.0 pmol/L in PSN632408-treated mice, 35.9±8.6 pmol/L in sitagliptin-treated mice, and 44.2±10.5 pmol/L in PSN632408 plus sitagliptin-treated mice (p<0.01, all treated groups vs. control). The plasma GLP-1 level in PSN632408 plus sitagliptin-treated mice was significantly higher than in PSN632408-treated mice (p<0.05), and slightly but not significantly higher than the level in sitagliptin-treated mice.

### PSN632408 and Sitagliptin Improved Oral Glucose Tolerance

**Figure 3:**
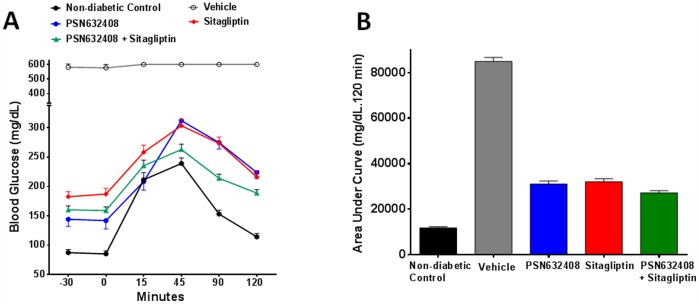
(A) OGTT in non-diabetic control mice and diabetic mice treated with vehicle, and those that achieved normoglycemia after 7 weeks treatment with PSN632408, sitagliptin, or PSN632408 and sitagliptin combination (n = 5 mice per group). Blood glucose levels in mice treated with PSN632408 and sitagliptin combination were significantly lower than in mice treated with PSN632408 alone at 45 minutes and at 90 minutes (p<0.01) and in mice treated with sitagliptin alone at 90 minutes (p<0.01). **(B)** Blood glucose AUC_0–120_ in mice treated with PSN632408, sitagliptin, or PSN632408 and sitagliptin combination was significantly lower than in mice treated with vehicle (P<0.01).

OGTT was performed in mice with diabetes reversal after treatment. Vehicle-treated mice showed blood glucose levels >600 mg/dL post-glucose administration that did not decline throughout the 120-minute time period, whereas PSN632408 and sitagliptin combination treatment significantly increased glucose clearance at 45 minutes and at 90 minutes compared with PSN632408 alone (p<0.01) and significantly increased glucose clearance at 90 minutes compared with sitagliptin alone (p<0.01) (Figure 3A). Furthermore, blood glucose AUC for 120 min (AUC_0–120_) was 84,893±1,758 mg/dL in vehicle-treated mice, 31,113±1335 mg/dL in PSN632408-treated mice, 32,104±1,308 mg/dL in sitagliptin-treated mice, and 27,207±941 mg/dL in PSN632408 plus sitagliptin-treated mice (p<0.05 for all groups vs. vehicle) (Figure 3B).

### PSN632408 and Sitagliptin Treatment Increased β-Cell Mass

**Figure 4:**
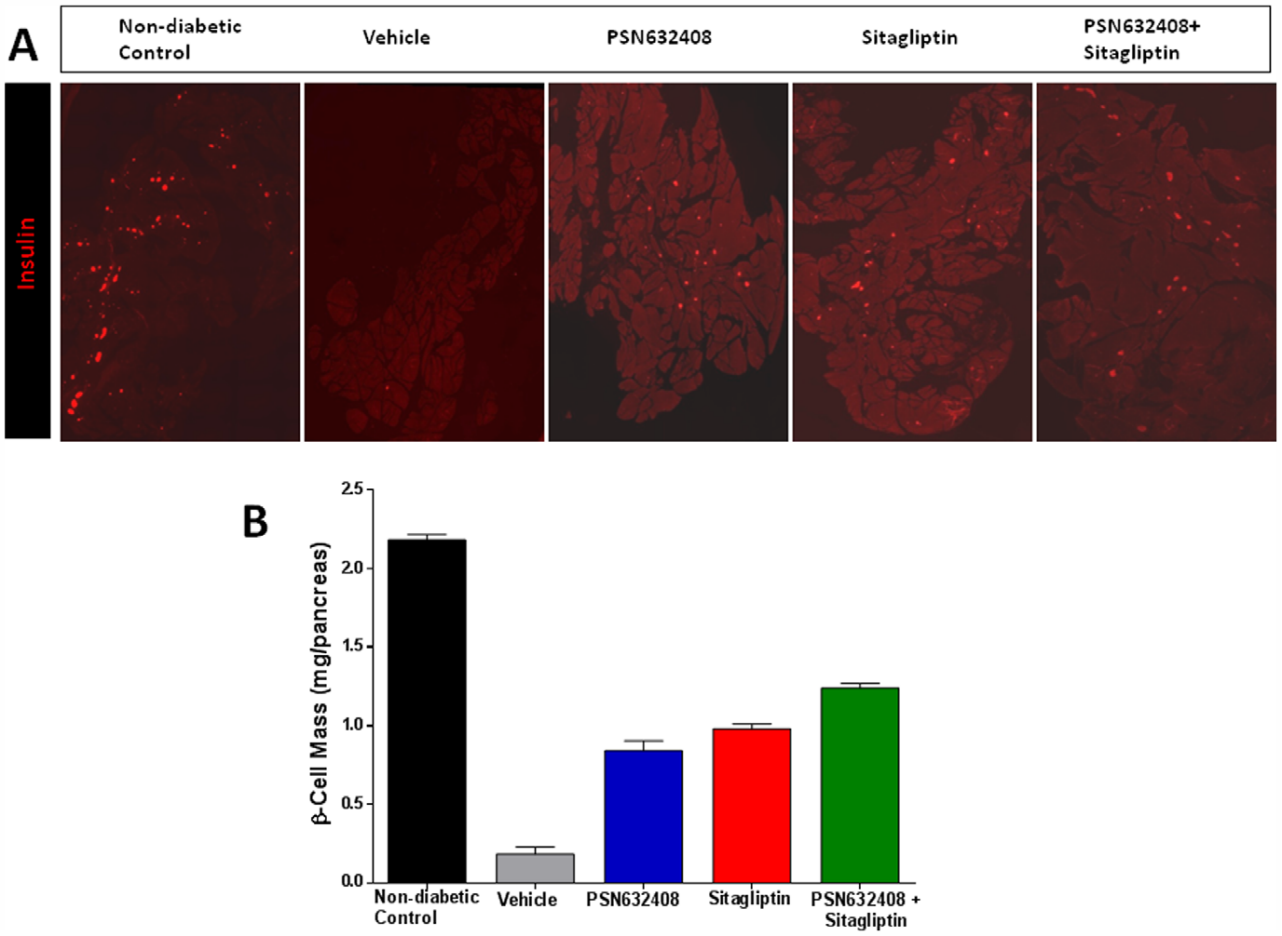
(A) Confocal images represent insulin (red) immunostained pancreas sections from diabetic mice treated with vehicle, PSN632408, sitagliptin, or PSN632408 plus sitagliptin. **(B)** Quantification of β-cell mass in the pancreases of non-diabetic control mice and diabetic mice with restored normoglycemia after 7 weeks of treatment with PSN632408, sitagliptin, or PSN632408 and sitagliptin combination (3 to 4 mice per group). β-Cell mass was significantly higher in mice treated with PSN632408 and sitagliptin combination than in mice treated with PSN632408 alone or sitagliptin alone (P<0.05).

In vehicle-treated diabetic mice, only a few insulin positive cells were detected in the pancreas sections. However, many insulin positive cells were seen in the pancreas sections of non-diabetic control mice and diabetic mice that achieved normoglycemia after treatment with PSN632408, or sitagliptin, or PSN632408 combined with sitagliptin (Figure 4A). Pancreatic β-cell mass in vehicle-treated diabetic mice (0.18±0.04 mg) was significantly reduced compared with non-diabetic control mice (2.18±0.03 mg, p<0.01), whereas treatment with PSN632408 and sitagliptin either alone or in combination significantly increased β-cell mass (Figure 4B). The pancreatic β-cell mass was 0.84±0.06 mg in PSN632408-treated mice, 0.98±0.03 mg in sitagliptin-treated mice, and 1.24±0.02 mg in PSN632408 plus sitagliptin-treated mice (p<0.01 for all groups vs. vehicle). Also, β-cell mass in PSN632408 plus sitagliptin-treated mice was significantly higher than in mice treated with either PSN632408 alone, or sitagliptin alone (p<0.05). Pancreatic β-cell mass was restored to 39% in PSN632408-treated mice, 45% in sitagliptin-treated mice, and 56% in PSN632408 plus sitagliptin-treated mice, compared with non-diabetic control mice (set at 100%).

### PSN632408 and Sitagliptin Treatment Stimulated β-Cell Replication

**Figure 5:**
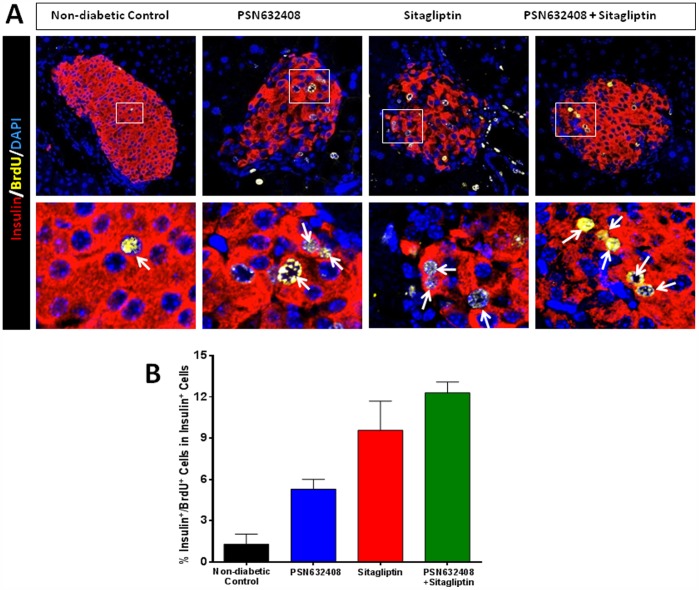
(A) Immunostaining of insulin (red), BrdU (yellow), and DAPI (blue) in pancreas sections from BrdU treated non-diabetic control mice and diabetic mice with restored normoglycemia after 7 weeks of treatment with PSN632408, sitagliptin, or PSN632408 combined with sitagliptin. White arrows indicate insulin/BrdU/DAPI co-positive cells. **(B)** The percentage of insulin/BrdU co-positive cells among total insulin positive cells was significantly higher in mice treated with PSN632408 and sitagliptin combination than in mice treated with PSN632408 alone (p<0.01, n = 4 to 6 mice per group).

**Figure 6:**
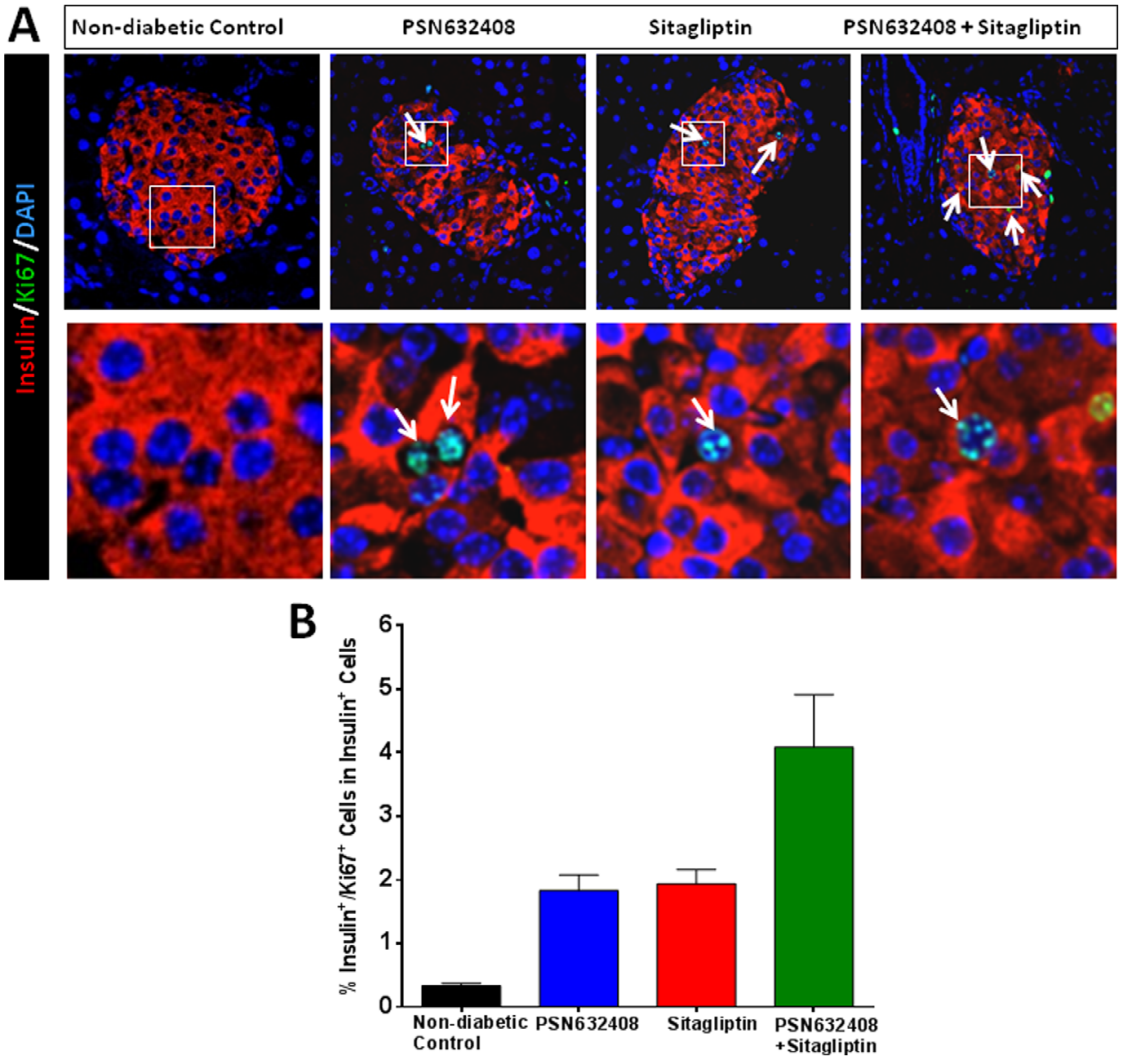
(A) Immunostaining of insulin (red), Ki67 (green), and DAPI (blue) in pancreas sections from non-diabetic control mice and diabetic mice with restored normoglycemia after 7 weeks treatment with PSN632408, sitagliptin, or PSN632408 combined with sitagliptin. White arrows indicate insulin/Ki67/DAPI co-positive cells. (B) The percentage of insulin/Ki67 co-positive cells among total insulin positive cells was significantly higher in mice treated with PSN632408 and sitagliptin combination than in mice treated with PSN632408 alone or sitagliptin alone (p<0.01, n = 4 to 6 mice per group).

Since vehicle-treated diabetic mice only have a few remaining β cells, non-diabetic control mice were given BrdU daily for 7 weeks as controls. Small numbers of insulin/BrdU co-positive cells were found in the pancreases of control mice; however, many more insulin/BrdU co-positive cells were seen in mice treated with PSN632408, sitagliptin, or PSN632408 and sitagliptin combination (Figure 5A). The percentage of insulin/BrdU co-positive cells was significantly higher in PSN632408-, sitagliptin-, and PSN632408 plus sitagliptin-treated mice than in non-diabetic control mice (Figure 5B). The percentage of insulin/BrdU co-positive cells was significantly higher in mice treated with PSN632408 and sitagliptin combination (12.3±0.8%) than in PSN632408-treated mice (5.3±0.7%, p<0.01), although it was not significantly higher than in sitagliptin-treated mice (9.57±2.9%, p>0.05). To further investigate β-cell replication, we performed immunostaining for Ki67, a marker for cellular replication that is incorporated during all active phases of the cell cycle. More insulin/Ki67 co-positive cells could be seen in the pancreases of mice treated with PSN632408, sitagliptin, or PSN632408 combined with sitagliptin (Figure 6A). The percentage of insulin/Ki67 co-positive cells was 4.1±0.8% in PSN632408 plus sitagliptin-treated mice and this was significantly higher than in either PSN632408-treated mice (1.8±0.2%) or sitagliptin-treated mice (1.9±0.2%, p<0.05) (Figure 6B). Thus, although treatment with PSN632408 alone or sitagliptin alone could stimulate β-cell replication, treatment with PSN632408 and sitagliptin combination was significantly better than either alone.

### PSN632408 and Sitagliptin Treatment Induced β-Cell Neogenesis

**Figure 7:**
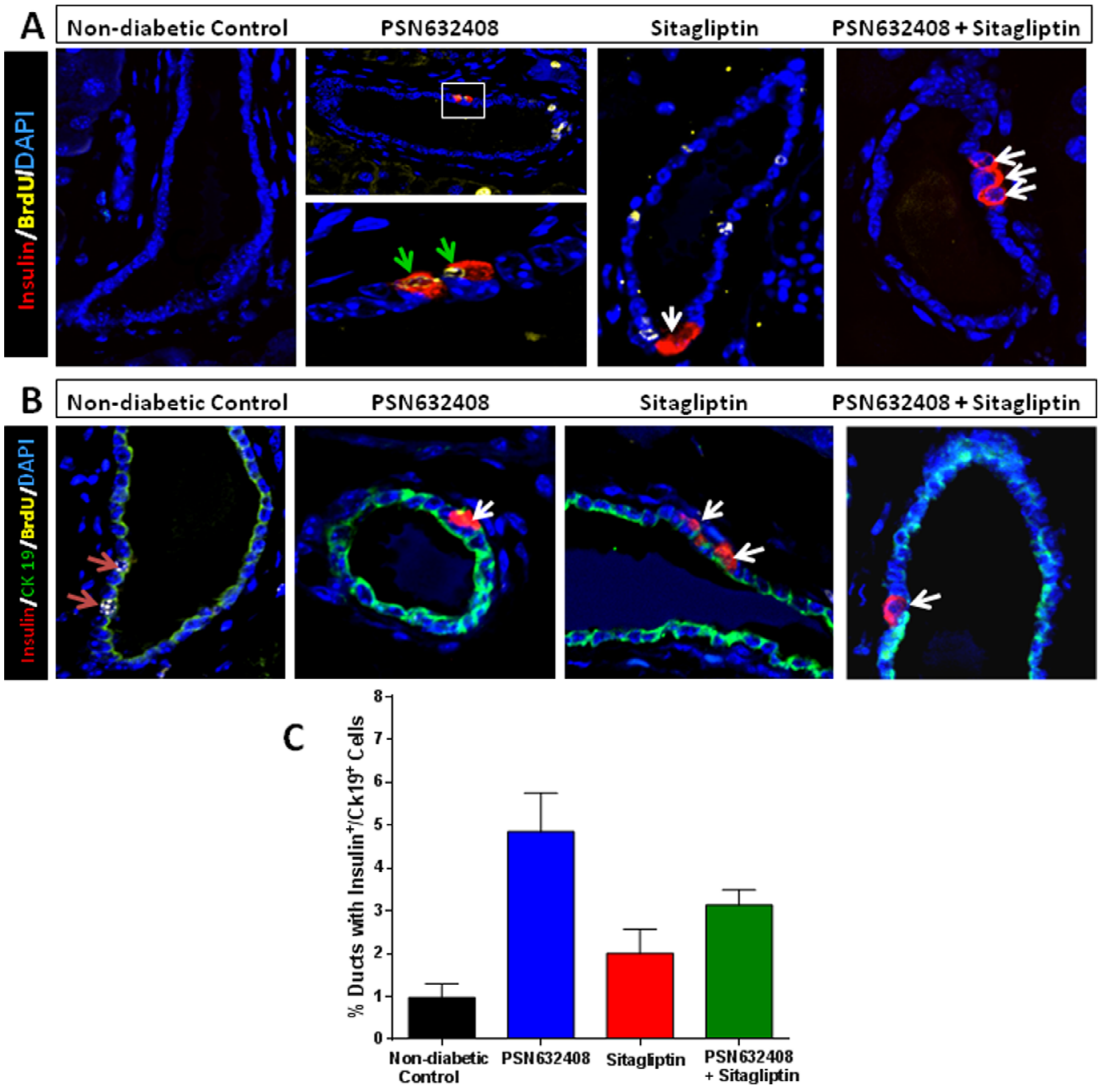
(A) Immunostaining of insulin (red), BrdU (yellow) and DAPI (blue) in pancreas sections from BrdU-treated non-diabetic control mice and diabetic mice with restored normoglycemia after 7 weeks treatment with PSN632408, sitagliptin, or PSN632408 combined with sitagliptin. White arrows indicate insulin/DAPI co-positive pancreatic duct cells. Green arrows indicate insulin/BrdU/DAPI co-positive pancreatic duct cells. **(B)** Immunostaining of insulin (red), CK19 (green), BrdU (yellow), and DAPI (blue) in pancreas sections from these mice. Red arrow indicates CK19/BrdU co-positive pancreatic duct cells. White arrows indicate insulin/CK19 co-positive pancreatic duct cells. **(C)** The percentage of ducts with insulin/CK19 co-positive cells was significantly increased in mice treated with PSN632408 alone or PSN632408 and sitagliptin combination compared with non-diabetic control mice (p<0.05, n = 3 to 6 mice per group).

Since pancreatic duct cells or precursors in the ducts may be involved in β-cell regeneration, we performed immunostaining for insulin and CK19, a duct cell marker, and examined the ducts in the pancreases. Although insulin positive cells and insulin/CK19 co-positive cells could be seen in non-diabetic control mice, more insulin positive cells and insulin/CK19 co-positive cells were found in the ducts of pancreases in mice treated with PSN632408, sitagliptin, or PSN632408 combined with sitagliptin (Figure 7A and B). The percentage of ducts with insulin/CK19 co-positive cells in the pancreas of non-diabetic control mice (1.0±0.3%) was significantly increased in PSN632408-treated mice (4.9±0.9%) and in PSN632408 plus sitagliptin -treated mice (3.13±0.35%, p<0.05, vs. control), but not in sitagliptin-treated mice (2.0±0.6%, P>0.05, vs. control) (Figure 7C). These findings suggest that 7 weeks of PSN632408 treatment alone or in combination with sitagliptin could stimulate β-cell neogenesis from cells lining the pancreatic ducts.

### PSN632408 and Sitagliptin Treatment Increased α-Cell Replication but not Exocrine Cell Replication

**Figure 8:**
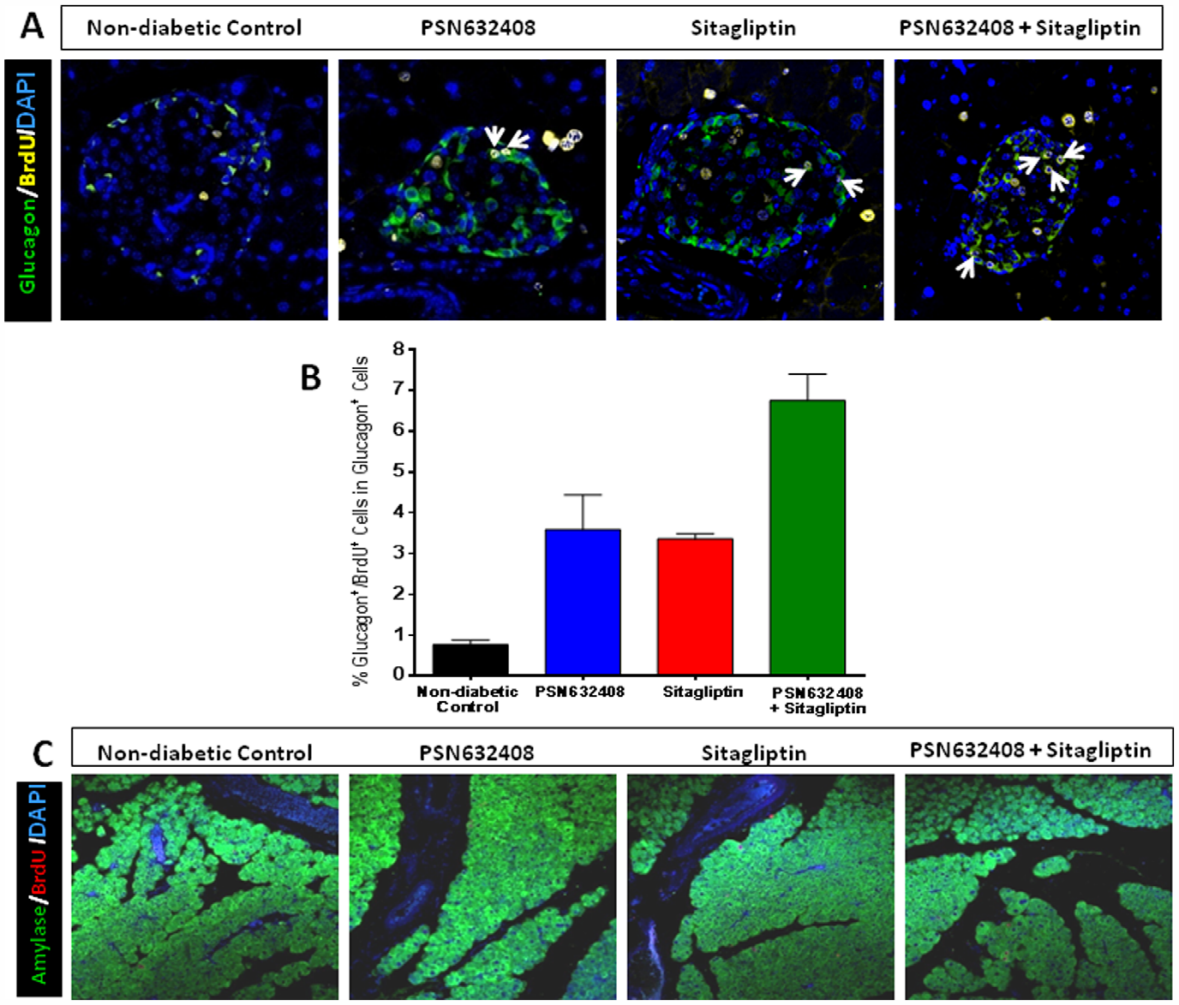
(A) Immunostaining of glucagon (green), BrdU (yellow), and DAPI (blue) in pancreas sections from BrdU-terated non-diabetic control mice and diabetic mice with restored normoglycemia after 7 weeks treatment with PSN632408, sitagliptin, or PSN632408 combined with sitagliptin. White arrows indicate glucagon/BrdU/DAPI co-positive cells. **(B)** The percentage of glucagon/BrdU/DAPI co-positive cells among total glucagon positive cells was significantly increased in mice treated with PSN632408 and sitagliptin combination compared with mice treated with PSN632408 alone or sitagliptin alone (p<0.05, n = 3 to 5 mice per group). **(C)** Immunostaining of amylase (green), BrdU (red), and DAPI (blue) in pancreas sections from BrdU-treated non-diabetic control mice and diabetic mice with restored normoglycemia after 7 weeks treatment with PSN632408, sitagliptin, or PSN632408 combined with sitagliptin.

Since islets are comprised of 15–20% glucagon-producing α cells, we sought to determine α-cell replication by determining glucagon/BrdU co-positive cells in islets. Interestingly, more glucagon/BrdU co-positive cells were found in mice treated with PSN632408, sitagliptin, or PSN632408 combined with sitagliptin than in non-diabetic control mice (Figure 8A). The percentage of glucagon/BrdU co-positive cells was significantly higher in PSN632408 plus sitagliptin-treated mice (6.8±0.6%) than in either PSN632408-treated mice (3.6±0.9%) or sitagliptin-treated mice (3.4±0.1%, p<0.05) (Figure 8B). Exocrine cell replication was evaluated by determining amylase/BrdU co-positive stained cells. A few replicating acinar cells were detected in non-diabetic control mice and in mice treated with PSN632408, sitagliptin, or PSN632408 combined with sitagliptin (Figure 8C) and there were no significant differences between control and treated mice (data not shown).

## Discussion

In this study, we examined the efficacy of combining a GPR119 agonist (PSN632408) with a DPP-IV inhibitor (sitagliptin) in C57BL/6 mice with STZ-induced diabetes. PSN632408 and sitagliptin combination treatment was significantly better at restoring normoglycemia than either agent given alone after 7 weeks of treatment. Hyperglycemia persisted in all vehicle-treated mice and these mice experienced significant reduction in body weights, whereas mice treated with PSN632408, sitagliptin, or the combination did not experience any decrease in body weight (data not shown). Treated mice with restored normoglycemia were further evaluated with an OGTT for their glucose-handling potential. PSN632408 and sitagliptin combination treatment significantly improved glucose tolerance as evidenced by increased glucose clearance.

Numerous studies in humans demonstrate the chronic effects of the DPP-IV inhibitor, sitagliptin to increase bioactive GLP-1 levels [8], [18], [19]. In a previous study, we found that the GPR119 agonist, PSN632408 treatment can also increase the plasma GLP-1 level in mice [17]. In the present study, we found that PSN632408 and sitagliptin combination treatment significantly increased plasma GLP-1 levels more than PSN632408 treatment alone. Our data are consistent with an earlier study which showed that combining a GPR119 agonist with a DPP-IV inhibitor is significantly better than either treatment alone on increasing plasma GLP-1 levels and improving oral glucose tolerance [12].

In our previous studies, we found that a DPP-IV inhibitor can stimulate pancreatic β-cell replication *in* *vivo* in nonobese diabetic (NOD) mice [20], and PSN632408 can stimulate β-cell replication *in vivo* and improve pancreatic islet graft function in mice with STZ-induced diabetes [17]. In this study, we found that PSN632408 and sitagliptin treatment alone or in combination could increase pancreatic β-cell mass. Our findings of increased β-cell mass, roughly in proportion to the increase in plasma GLP-1 levels in treated mice, is consistent with the report that increasing GLP-1 in DPP-IV-deficient mice is associated with enhanced β-cell survival after STZ injury [21]. Also, DPP-IV inhibition can preserve pancreatic β-cell mass and function by increasing the number of insulin positive β-cells in islets of mice with type 2 diabetes [22]. Using a STZ-induced diabetic mouse model, a recent study showed that a novel DPP-IV inhibitor can ameliorate diabetes by increasing β-cell replication and neogenesis [23]. Another study also showed that a novel, potent, specific and substrate-selective DPP-IV inhibitor can improve glycemic control and β-cell damage [24]. The restorative effects of those DPP-IV inhibitors following STZ injury on pancreatic β-parameters were overall consistent with our DPP-IV inhibitor, sitagliptin treatment. Furthermore, we found that combining a GPR119 agonist with a DPP-IV inhibitor is significantly better than either alone. Because GPR119 is expressed on pancreatic β-cells as well as intestinal L cells, it is possible that PSN632408 could have improved blood glucose levels and increased pancreatic β-cell mass by a direct action on β-cells and/or by stimulating GLP-1 production by intestinal L cells. These alternatives might be answered by using GLP-1 receptor knockout mice.

Multiple factors may contribute to the effects on pancreatic β-cell mass including β-cell regeneration (replication and neogenesis), hypertrophy and apoptosis. To assess β-cell regeneration in these mice, we continuously labeled mice with BrdU to track replicating cells and measured the proliferation rates in terms of percentage of insulin and BrdU co-positive cells in total islet β-cells. We found that PSN632408 and sitagliptin combination treatment significantly increased the numbers of replicating β-cells compared with vehicle or PSN632408 treatment alone. It has been suggested that BrdU incorporation is associated with a DNA damage response, not replication, in human pancreatic β-cells [25]. Therefore, we did not solely rely upon BrdU incorporation as evidence of β-cell replication. We used Ki67, a cellular marker for replication, to further determine β-cell replication. Ki67 is strictly associated with cell replication and is expressed during all phases of the cell cycle tracking active dividing cells. Although Ki67 staining would have identified the cells undergoing cell division during the last fraction of the treatment period, our results corroborated a similar trend observed with insulin and BrdU staining. Using insulin and Ki67 staining as reliable evidence of β-cell replication, we found that treatment with PSN632408 alone or sitagliptin alone could stimulate β-cell replication; however, PSN632408 and sitagliptin combination was significantly better than either alone. Whether the replication of these β-cells was from self-renewal of mature β cells or was from specialized progenitors in islets needs to be further investigated.

There is compelling evidence supporting β-cell neogenesis from precursors/stem cells in the ductal epithelium of the pancreas as a mechanism of β-cell regeneration in several diabetic models [26], [27], [28]. Exendin-4, a GLP-1 analogue, has been shown to stimulate not only β-cell replication, but also β-cell neogenesis [29]. In this study, we observed insulin positive cells located in the epithelial cell lining of pancreatic ducts. These insulin positive cells lining ducts were further confirmed to be exocrine duct cells, using CK-19, a ductal epithelial cell marker. Although, considerable animal to animal heterogeneity was observed across all treatment groups, mice treated with either PSN632408 alone or PSN632408 and sitagliptin combination showed significant increases in insulin/CK19 co-positive duct cells. We did not detect any glucagon positive cells in pancreatic ducts (data not shown). It has been suggested that mature pancreatic ducts could act as facultative stem cells or a pool of potential progenitors [26], [27]. Whether these newly differentiated cells are duct-derived progenitors or from another source should be further determined using lineage-tracing experiments. Also, monitoring PDX-1 expression at different stages of the treatment period may answer whether or not these ductal cells are contributing to islet neogenesis.

It is well known that β-cell replication strictly declines with age in mice [30], [31] and in humans [32], [33], [34], [35]. This phenomenon might be due to down regulation of key transcription factors and kinases implicated in β-cell mitosis [31]. In our studies, the mice were ∼10 weeks old after diabetes induction and the mice were treated for 7 weeks. These mice were not aged mice, hence the replicative pool of cells would be considered abundant. Interestingly, the rate of β-cell replication in the pancreas of STZ-induced diabetic mice treated with PSN632408 was lower than the rate of β-cell replication in islet grafts in STZ-induced diabetic mice treated with PSN632408 in our earlier study [17]. We do not know why PSN632408 could stimulate more β-cell replication in intact islet grafts. One possibility is that STZ demolished a lot of β-cells in the pancreas that have the capability to replicate. Another possible reason is that β-cells in intact islet grafts replicate more in a high glucose milieu, since almost all recipient mice had blood glucose levels >600 mg/dL before islet transplantation. Further studies are needed to determine whether β-cell replication is from self-renewal of mature β cells or from replication of specialized progenitors and whether different glucose levels affect β-cell replication in mice treated with PSN632408 and sitagliptin.

In addition to β cells, we found a more than 2-fold increase in replication of α-cells when mice were treated with PSN632408 or sitagliptin alone, and more than a 5-fold increase when treated with combination therapys; however, α-cell mass was not measured. Alpha-cell replication and elevated glucagon levels may aid in the formation of new β-cells, since pancreatic glucagon is required for β-cell formation and differentiation [36]. Also, the composition of α-cells increases in pancreatic islets of diabetic human patients and of animal models [37], [38]. Interestingly, α-cells can be converted into β-cells under conditions of extreme damage to β-cells [39]. Therefore, we cannot rule out the possibility that DPP-IV inhibitors or GPR119 agonists might play a role in α- to β-cell differentiation. There is some evidence for contribution of acinar cells in islet β-cell formation by transdifferentiation under specific conditions [40]; however, we did not find any increase in exocrine cell replication in any treatment group.

To the best of our knowledge, this is the first study demonstrating the effects of a GPR119 agonist along with a DPP-IV inhibitor on β-cell regeneration via both replication and neogenesis in a diabetic mouse model. Besides β-cell regeneration, other factors including prevention of β-cell apoptosis may have also contributed to the increase of β-cell mass. Pancreatic β-cell mass was remarkably improved by combination therapy, which offers a novel therapeutic strategy for treating diabetic patients with a low β-cell mass. As there are clear discrepancies between rodent and human β-cell regeneration capacity [32], [33], [34], [35], our future studies are aimed at evaluating the potential of these drug combinations on human islet regeneration by transplanting human islets from young and aged donors into immunodeficient mouse models. In addition, it will be interesting to further investigate the effect of combining a GPR119 agonist with a DPP-IV inhibitor on reversing autoimmune diabetes in NOD mice, because DPP-IV inihbitors alone can reverse new-onset diabetes in some NOD mice [20], [41].
